# Sea level: measuring the bounding surfaces of the ocean

**DOI:** 10.1098/rsta.2013.0336

**Published:** 2014-09-28

**Authors:** Mark E. Tamisiea, Chris W. Hughes, Simon D. P. Williams, Richard M. Bingley

**Affiliations:** 1 National Oceanography Centre, Joseph Proudman Building, 6 Brownlow Street, Liverpool L3 5DA, UK; 2 School of Environmental Sciences, University of Liverpool, Liverpool L69 3GP, UK; 3 Nottingham Geospatial Institute and Department of Civil Engineering, University of Nottingham, Nottingham NG7 2TU, UK

**Keywords:** geodesy, sea level, observing systems

## Abstract

The practical need to understand sea level along the coasts, such as for safe navigation given the spatially variable tides, has resulted in tide gauge observations having the distinction of being some of the longest instrumental ocean records. Archives of these records, along with geological constraints, have allowed us to identify the century-scale rise in global sea level. Additional data sources, particularly satellite altimetry missions, have helped us to better identify the rates and causes of sea-level rise and the mechanisms leading to spatial variability in the observed rates. Analysis of all of the data reveals the need for long-term and stable observation systems to assess accurately the regional changes as well as to improve our ability to estimate future changes in sea level. While information from many scientific disciplines is needed to understand sea-level change, this review focuses on contributions from geodesy and the role of the ocean's bounding surfaces: the sea surface and the Earth's crust.

## 1. Introduction

Sea level has a very significant place in science and society. The public perhaps most frequently consider sea level in relation to rapid changes and extreme events, such as those that occur during storm surges and tsunamis, or even the tides. Indeed, in the UK, as a seafaring nation, understanding tides has always been vital for port operations and safe navigation, with tidal observations the subject of an article in the first volume of *Philosophical Transactions* [1]. However, slower changes in sea level are also important for society. Given that past sea levels have been quite different from those of today, both higher and lower, it is important to understand where sea levels might be in the future. In particular, as the local sea level rises, the return periods for extreme events tend to decrease, increasing the risk to society. Sea level is even part of daily life, as the UK's height system, Ordnance Datum Newlyn, is based on the mean sea level at Newlyn from 1915 to 1921, and height systems in many other countries are similarly tied to tide gauge measurements.

Measuring sea-level change at one location over short periods, such as the diurnal and semi-diurnal time scales of tides, is not particularly challenging. However, relating two measurements in time or space, whether separated by 100 years or 10 000 km, requires that we have a common reference system that is stable. Given that the Earth is constantly deforming and that we would like to make measurements at the submillimetre level (see below), this is challenging. Geodesy, by using observations of changes in the Earth's shape, gravity field and rotation, is able to provide such a reference system. Thus, it plays a vital role in sea-level science [2].

Understanding sea-level change, though, requires input from a large number of disciplines [3]. The atmosphere provides the most important forcing of the ocean, directly affecting sea level, and thus observations of surface pressure, precipitation, winds and temperature are vital. For long-time-scale changes in the ocean mass, knowledge of mass loss from ice sheets and glaciers and water storage changes on land and in the atmosphere is essential. Changes in temperature and salinity are able to account for much of the spatial variation observed in sea level [4]. Argo is a global system of over 3500 instruments that sample the temperature and salinity of the ocean by descending to 2000 m and returning to the surface every 10 days. Argo has allowed for the most complete picture of this component of sea-level change [5], although admittedly, up to now, missing data from below 2000 m and the marginal seas and ice-covered regions.

While all of these components are necessary to understand sea-level change, this review focuses on different geodetic measurements of sea level and how they are interconnected. An important aspect of these measurements is the changing geoid and crustal deformation, which are frequently considered static in oceanography and thus ignored. For this reason, we refer to the geoid and the crust as the static boundaries in the discussion below. We also focus primarily on trends in the data, as assessing this long-term variability highlights the need for long and continuous observations.

Finally, this article resulted from a contribution from the Challenger Society for Marine Science Prospectus 2013 meeting, entitled ‘A prospectus for UK marine sustained observations’. Given that focus, many of the examples are drawn from the context of UK sea-level science. However, understanding sea-level change is a global problem, requiring global observing systems.

### (a) Oceanographic view

**Figure 1.**
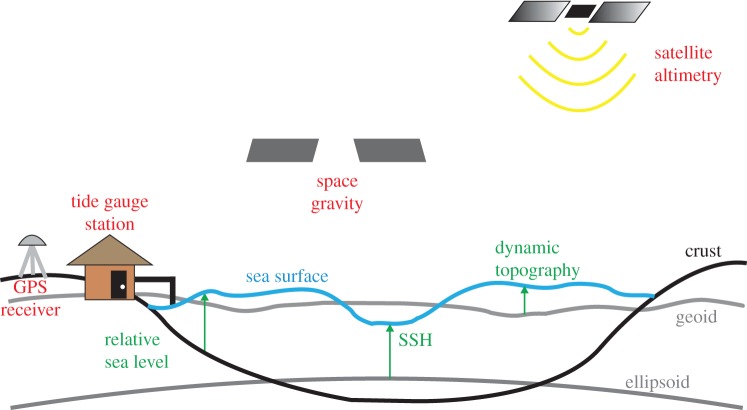
Simple schematic illustrating the relationship between sea surface height (SSH), the geoid, and dynamic topography. Included on the figure are representations of different components of the observing system and their respective measurement: GPS (or GNSS) for crustal deformation, satellite gravity for the geoid, altimetry for SSH and tide gauges for relative sea level. (Online version in colour.)

Before exploring the geodetic aspects of sea level, we should review how sea-level changes are viewed in oceanography. In the absence of dynamics or other external forcing, the surface of the ocean should lie on an equipotential of the Earth's gravity field, called the geoid. In the vast majority of ocean models and theoretical studies, this surface is represented by a constant value of the vertical coordinate *z*. It is important, therefore, to recognize that *z* is not simply a geometrical coordinate, but is defined by the Earth's gravity field. With the exception of global tide models, which explicitly account for solid Earth deformation and changes to the gravity field (loading and self-attraction), most ocean models assume both bathymetry and the *z*-coordinate to be fixed relative to the rotating Earth. The deviation of the ocean's surface, or the sea surface height (SSH), from the geoid is called dynamic (ocean) topography (figure 1). Winds, together with atmospheric pressure and surface fluxes of heat and freshwater, determine the dynamic topography (the response to tidal forces is usually considered separately).

When looking at how SSH changes in time, oceanographers typically assume that the geoid is constant and it is only the dynamic topography which is changing. This is generally a good approximation at periods of a few years or less, but long-term changes in the geoid will eventually become important. The dynamic topography is especially valuable because it defines the geostrophic current, which dominates the ocean flow outside frictional boundary layers at periods longer than a few days [6]. It is because of this relationship that satellite altimetry measurements of SSH variability have revolutionized our understanding of oceanography, particularly the role of mesoscale eddies, which act on typical length scales of 10 to a few hundred kilometres [7], and periods of about 10–200 days [8].

**Figure 2.**
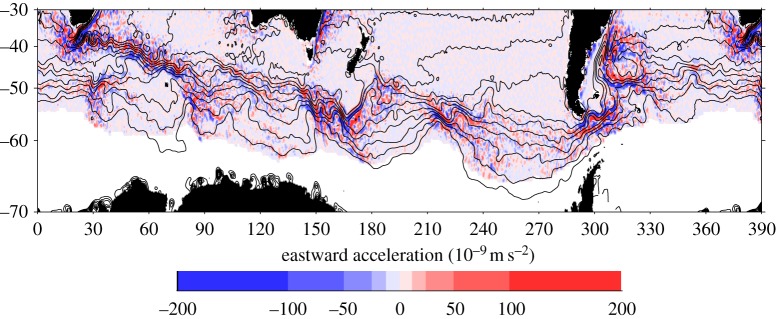
Eastward acceleration of the mean flow by time-dependent eddies, based on 13 years of satellite altimetry data (a dynamically passive, irrotational component has been removed). Contours representing intervals of 20 centimetres show mean dynamic topography based on the DTU10 [16,17] mean sea surface and the TUM2013C geoid [18], averaged over 0.25° blocks and with 25 km Gaussian smoothing applied.

The many oceanographic breakthroughs from altimetric measurements would require an extensive review article. However, a few examples include the first mapping of global Rossby wave speeds [9] and the recognition that such waves are swept to the east in the Antarctic circumpolar current [10,11]; the discovery of alternating zonal jets throughout much of the ocean [12]; the recognition that much of what had initially been thought of as Rossby waves is in fact nonlinear eddy variability, and the mapping of the eddy characteristics [13]; recognition of large-scale changes in the North Atlantic circulation related to the Meridional overturning circulation [14]; and unravelling of the complex interactions between basin-scale flows, climate modes, Rossby waves and mesoscale eddies in the North Pacific Ocean ([15] and references therein). All of these observations have stimulated theoretical development and improved understanding of the global ocean circulation. Figure 2 (adapted from [19]) shows an example of what can be learned about eddy-mean flow interaction from satellite altimetry. The shading represents the eastward acceleration of the mean flow owing to momentum fluxes carried by time-dependent eddies (measured using altimetry alone), whereas the contours of mean dynamic topography illustrate the mean flow (for which a geoid measurement is also needed). It had been thought that eddies radiated out from jets would tend to exert an eastward acceleration on the jets, but the observations showed a more complicated relationship, indicating that the simple theory is too idealized to apply to the real Southern Ocean, and suggesting that the interaction of eddies with topography is important.

Going back to figure 1, the problem with the typical oceanographic viewpoint that all of the changes in SSH are due to changes in dynamic topography is that neither the geoid nor the ocean's crust is stationary, and SSH changes can be driven by their motion. These geoid and crustal changes, while typically small compared with the tens of centimetre changes driven by oceanographic processes on shorter time scales, become increasingly important as the time scale of interest increases and the size of the dynamic ocean signal decreases. This is exemplified by sea-level change, as one would like to measure changes over years to decades to centuries to an accuracy of a fraction of a millimetre per year. In addition, coastal cities around the world are built upon the Earth's crust. Thus, even if the water volume of the ocean was constant, a city will be inundated if the local region is subsiding. (Tide gauges directly measure this relative sea-level change, whereas an independent measure of the crustal motion is needed to convert a measurement from a satellite to a relative sea-level measurement, as described below.) Therefore, to understand observed long-term sea-level changes, particularly at the vulnerable coastlines, it becomes essential to account for both the crustal motion and geoid changes.

### (b) Changes to the static boundaries

**Figure 3.**
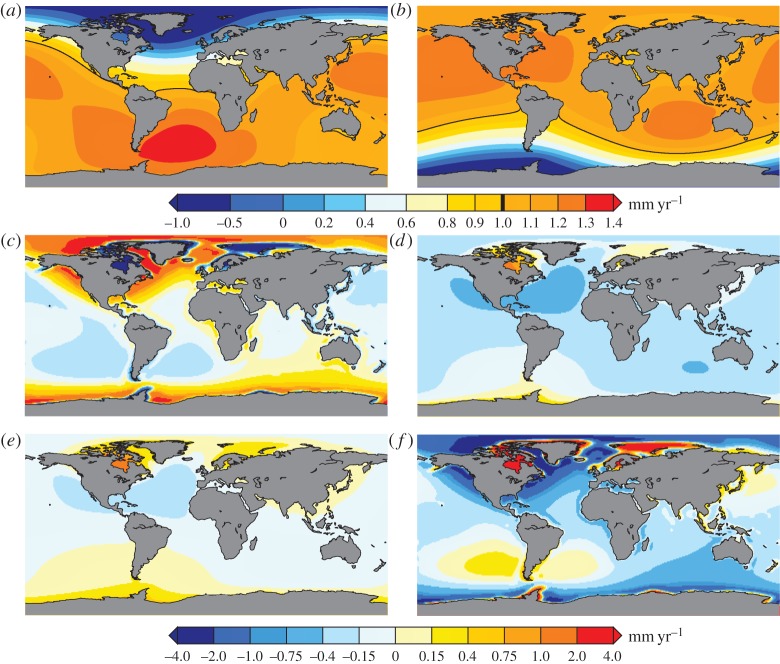
(*a*,*b*) Fingerprint of relative sea-level change caused by a mass loss scenario equivalent to 1 mm per year of globally averaged sea-level rise from (*a*) Greenland and (*b*) West Antarctica. The 1 mm per year contour is marked with a black line in these panels and in the colour bar. These results assume that mass loss occurs rapidly compared with the time over which the mantle would flow. Under this assumption, these maps can be scaled by the actual contributions from each region. (From fig. 4*a*,*b* in [33]. Copyright © 2011 The Oceanography Society, Inc.). (*c*–*f*) An example GIA model predication of change in (*c*) relative sea level (tide gauges), (*d*) geocentric sea level (altimetry), (*e*) geoid change (GRACE) and (*f*) crustal motion (GNSS) [34]. These results use a modified version [34] of the ICE-5G ice model and VM2 Earth model [35].

Many processes can drive the change to the geoid and crust, on a wide range of time scales. One of the most-modelled examples is the effect of mass loss from ice sheets and glaciers on sea-level change. (Other processes, such as earthquakes, can have large regional effects, but the global impact on sea-level estimates is not as extensively modelled [20,21].) The mass component of the associated freshwater flux into the oceans would be redistributed globally on a subweekly time scale by barotropic waves [22–24]. (Sea-level variations are also driven by the resulting temperature and salinity perturbations, but these take much longer to redistribute [25].) However, this does not imply that the rapid mass redistribution would be uniform [26–30]. The redistribution of mass drives crustal motion and gravity changes, which introduce long length-scale variations into the sea-level change. Initially, the response of the Earth is nearly elastic, i.e. deformation occurs as soon as the surface or potential load changes and recovers as soon as the load returns to its initial state. A demonstration that the Earth does respond on very short time scales to changing applied forces is the solid Earth's centimetre-level tidal response [31,32]. Figure 3*a*,*b* shows model predictions for tide gauge measurements of sea level measured relative to the crust as a result of mass loss from Greenland and West Antarctica [33]. In regions where the mass loss occurs, whether owing to sublimation, melting or discharge, the gravitational attraction is reduced, causing the geoid to lower near the ice sheet. Thus, an altimeter would observe a sea-level fall. However, this fall is even larger for a tide gauge measurement in the region owing to the resulting crustal uplift. Owing to this near-field relative sea-level fall and a need for this to be balanced in the global average, far-field sea level in some regions must rise by more than average.

After a period of time, which varies from region to region, the Earth's response ceases to be purely elastic and the regional mass loss will cause flow in the Earth's crust and mantle. Thus, past changes in ice cover, particularly in the most recent glacial period which peaked over 19 000 years ago, still drive sea-level changes observed today. This long-term response of the Earth is referred to as glacial isostatic adjustment (GIA). Figure 3*c*–*f* shows one particular model prediction of the GIA effects on tide gauges (relative sea level), altimetry (geocentric sea level or the sea surface), the geoid as measured by satellites (i.e. a specified geopotential surface) and crustal motion respectively [34]. The largest features on the map are uplift (and relative sea-level fall) under the centres of the former ice sheets and subsidence (and relative sea-level rise) in the peripheral regions where the forebulge is collapsing. Note that the crustal motion has much shorter length-scale variations than the altimetry prediction. Thus, it is the crustal motion that contributes this feature to the tide gauge prediction, as figure 3*d* minus figure 3*f* gives figure 3*c*. It should also be noted that in subduction zones, e.g. Alaska [36] and Chile [37], flow in the Earth's crust and mantle can occur more quickly, making the recent decadal history of ice mass change more important and removing the time-scale separation of the long-term GIA response and the elastic response described in the previous paragraph.

The GIA example demonstrates that the different methods of assessing sea-level change actually measure different aspects of this problem. By investigating the spatial variability of the different data types, one may be able to extract additional information on the cause of sea-level change. In addition, as we discuss later, each of the different observation systems has limitations. Thus, as we illustrate, the combination of techniques allows the most complete insight into the causes of global sea-level rise and its spatial variability.

### (c) Geodetic contributions to observing sea level

Geodesy is the science of using observations of changes in the Earth's shape, gravity field and rotation to better understand the Earth and the processes occurring upon it. Geodesy is essential for understanding changes in sea level for a number of reasons. The most immediate is that the static changes discussed in §1*b*, whether they are due to water motion on the Earth's surface or flow within the mantle, are directly measured by these observations. However, geodesy also provides a reference system for use in altimetry and tide gauge measurements. In order to compare these observations from one year to another, or from one location to another, it is essential that the realization of this reference system is stable and accurate.

Observations are the basis for the realization of the reference frame. (Unfortunately, discussions of the geodetic observations and associated services typically involve a large number of abbreviations, which are, in some cases, better known than the actual name. We include a list in table 1.) The geometrical (positional) and rotational measurements are integrated into the International Terrestrial Reference Frame (ITRF), with the most recent release being ITRF2008 [38], which is coordinated by the International Earth Rotation and Reference Systems Service (IERS) [39]. One of the foundational observations for ITRF is very long baseline interferometry (VLBI), which uses the difference in simultaneous observations of quasars to establish Earth orientation parameters and scale, and the observations are coordinated by the International VLBI Service for Geodesy and Astrometry (IVS) [40]. The International Laser Ranging Service (ILRS) [41] coordinates satellite laser ranging (SLR), which measures the distance to orbiting satellites from a network of stations to establish the position of Earth's geocentre as well as contributing to the scale of the ITRF. Both VLBI and SLR have fairly sparse networks and thus ties between VLBI and SLR, as well as densification of the deformation observation, are provided by two additional systems: global navigation satellite systems (GNSSs—with the Global Positioning System, GPS, being one component) and Doppler Orbitography and Radiopositioning Integrated by Satellite (DORIS). The products generated from these observations are created by the International GNSS Service (IGS) [42] and International DORIS Service (IDS) [43].

**Table 1.** List of abbreviations used throughout the paper.

abbreviation	description
AG	absolute gravity
BIGF	NERC British Isles continuous GNSS Facility
CF	centre of figure of the solid Earth
CM	centre of mass of the whole Earth system
DORIS	Doppler Orbitography and Radiopositioning Integrated by Satellite
EA	Environment Agency
EOF	empirical orthogonal function
GCN	GLOSS Core Network
GGOS	Global Geodetic Observing System
GIA	glacial isostatic adjustment
GLOSS	Global Sea Level Observing System
GNSS	global navigation satellite system
GOCE	Gravity Field and Steady-state Ocean Circulation Explorer
GPS	Global Positioning System
GRACE	Gravity Recovery And Climate Experiment
IDS	International DORIS Service
IERS	International Earth Rotation and Reference Systems Service
IGS	International GNSS Service
ILRS	International Laser Ranging Service
IOC	Intergovernmental Oceanographic Commission
ITRF	International Terrestrial Reference Frame
IVS	International VLBI Service for Geodesy and Astrometry
IUGG	International Union of Geodesy and Geophysics
MERIT	Monitor Earth Rotation and Intercompare the Techniques
NERC	Natural Environment Research Council
NSGF	NERC Space Geodesy Facility
OBP	ocean bottom pressure
PSI	persistent scatterer interferometry
PSMSL	Permanent Service for Mean Sea Level
RLR	revised local reference
SLR	satellite laser ranging
SONEL	Système d’Observation du Niveau des Eaux Littorales
SAR	synthetic-aperture radar
SSH	sea surface height
SWOT	Surface Water, Ocean Topography
TIGA	GNSS Tide Gauge Benchmark Monitoring
VLBI	very long baseline interferometry
VLIZ	Flanders Marine Institute

The dramatic improvement in the measurement of the long-wavelength component of the geoid has led to the ability to directly derive the dynamic topography. Two recent satellite missions are responsible for this improvement: the Gravity Field and Steady-state Ocean Circulation Explorer (GOCE) [44] and the Gravity Recovery And Climate Experiment (GRACE) [45]. While not discussed further in this paper, the short-wavelength field can also be important, particularly along the coasts. Because the short-wavelength field is primarily constrained by terrestrial, marine and aerial measurements, this component of the gravity field is known better over well-observed regions of land than over the oceans. The global model that incorporates the most complete set of this information (though it does not include GOCE data) is the Earth Gravitational Model 2008 (EGM2008) [46].

As one of its activities, the Global Geodetic Observing System (GGOS) [47] facilitates dialogue between the geodetic services with the goal of improving the reference frame. The accuracy requirements for sea-level measurements are one of the main scientific drivers for these improvements. Because we want to understand sea-level change at the 1 mm per year level, being able to measure to the 0.1 mm per year level would be ideal [2]. How well we achieve this goal depends upon the type of observation, the variability of the time series and the temporal and spatial averaging. For example, instantaneous measurements from tide gauges or altimetry are only accurate to the centimetre level at best, but averaging can allow us to obtain better estimates. In the following, we indicate where possible the levels of uncertainties in the measurements.

In each of the sections following, we discuss the different observation systems, and their contributions to understanding sea-level change. We start by reviewing the two measurements commonly associated with sea level, tide gauges and satellite altimetry, and then move onto the measurements of the geoid and the crust. As is demonstrated in §1*b*, different observations measure different aspects of sea level, and thus should not be expected to give the same answer. While this is easily discussed in terms of model results, the differences are difficult to extract from the data given the level of uncertainty and variability. Some of this uncertainty is systematic owing to limitations on the ability to establish a stable realization of the reference system. The following discussion and thoughts on future integration of the different techniques should highlight the need for long-term observations and the continued need for improvements to the system.

## 2. Tide gauge data

**Figure 4.**
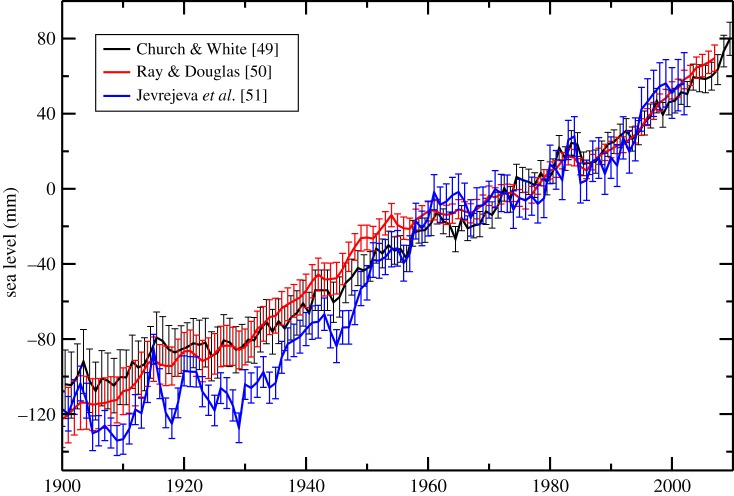
Three reconstructions [49–51] of sea-level rise during the twentieth century derived from tide gauge data. Each reconstruction uses different methods to combine the tide gauge data, as well as different selection criteria for choosing which records are used. The mean over the period 1960–1989 (30 years) was removed from each time series, as there can be an arbitrary offset in the reconstructions.

The longest instrumental time series of sea-level observations (indeed, of any oceanographic variable) come from tide gauges. Tide gauges, as the name implies, were originally used to record the tides for port operations. Owing to their ability to record observations at high frequencies, the data have also been used to study a wide range of processes, such storm surges and tsunamis, which have a large impact on society. However, it is the long time series of mean sea-level data, available from the Permanent Service for Mean Sea Level (PSMSL) [48], that provide the primary evidence of globally averaged sea-level rise during the twentieth century. Figure 4 shows three reconstructions [49–51] of global sea-level rise using very different techniques, from using empirical orthogonal functions extracted from altimetry to more direct averaging of the tide gauge time series, to derive the global values from the PSMSL dataset. While there are notable differences between the curves, they show many similar features, such as a faster rate from the 1930s to the 1950s [49–51], and an increased rate since the 1990s. Given that two of the reconstructions use a spatially constant empirical orthogonal function (EOF), or EOF_0_, in order to better represent the global average sea-level rise [49,50], this effectively leads to only using the altimetry data near the tide gauge sites, which may explain the similarity in the results [52].

The effort to collect the time series of mean sea level was initiated at the 1933 conference of the International Union of Geodesy and Geophysics (IUGG). Finnish Professor Rolf Witting suggested forming a mean sea-level committee to collect the data, because ‘for the solution of a complex of geophysical problems, data regarding sea level and its changes are of great importance’ [53]. Much of the work was conducted by Professor Joseph Proudman of the Liverpool Tidal Institute, who was then secretary of the committee. In 1940, he published the first compilation in the *Publications Scientifiques* series of the International Association for Physical Oceanography. Given this history, the PSMSL considers 1933 as its inaugural year.

**Figure 5.**
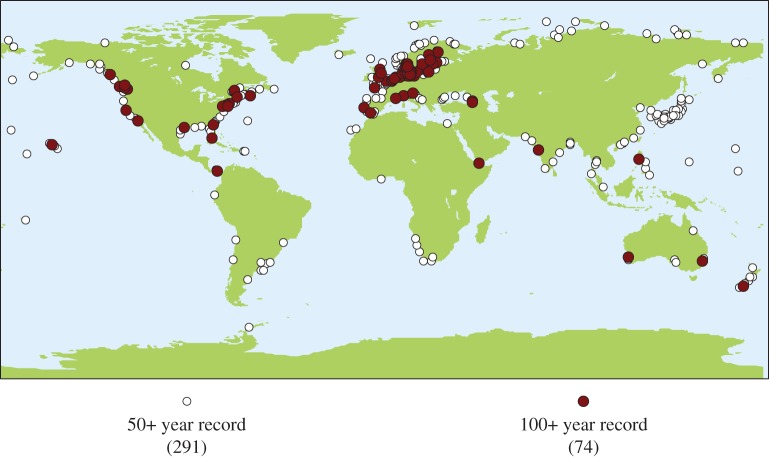
Tide gauge locations where the time series held by the PSMSL is greater than 50 years. This figure only includes gauges that have datum control (i.e. are in the RLR set), and thus can be used for long-term trend analysis. (Online version in colour.)

Since the publication of the initial collection of data, the PSMSL has continued its efforts and now holds data from over 2200 tide gauge locations and typically adds 1375 new station-years of data per year [48]. While this database provides the best long-term instrumental observations of sea-level change, there are limitations. In order to reduce the effects of decadal variability in the observations to obtain a more stable estimate of the trend, long time series are needed [54]. Unfortunately, nearly all of these long records are located in the Northern Hemisphere, with most in Europe (figure 5). This introduces greater uncertainty in establishing the global average rise. Given the importance of long records, there has been a concerted effort to recover historic tidal records, with more than 4000 station-years existing based on a recent global survey [55], and convert them to a digital form to allow analysis using modern techniques. A number of these studies have focused on extending modern tide gauge records in the Southern Hemisphere, such as Port Louis [56], Port Arthur [57] and Macquarie Island [58].

In addition, because many tide gauges are situated in locations that may experience very localized subsidence, as well as to facilitate the inevitable replacement of the gauge itself, all gauges must be levelled to local benchmarks in order to ensure the long-term stability of the time series. At the PSMSL, locations with this benchmark information are included in the revised local reference (RLR) set, which adjusts the tide gauge time series on the basis of the levelling information. Only slightly over 1400 of the stations in the PSMSL dataset are RLR.

Recognition of the importance of tide gauge data for oceanographic, climate and coastal sea-level research and the desire to improve the geographical distribution of a core network led to the establishment the Global Sea Level Observing System (GLOSS) in 1985 under the auspices of the Intergovernmental Oceanographic Commission (IOC). The GLOSS programme represents the global ocean observing system component with the largest level of international participation, with over 80 countries providing data to at least one of the data centres. While GLOSS has many subnetworks, the most recent implementation of the GLOSS Core Network (GCN) includes 290 stations [59]. GLOSS also has several data streams differentiated by their sampling frequency, level of quality control and availability delay (latency). These range from the monthly and annual means provided by the PSMSL with delays of 1 year or more to raw data streams (typically several minute averages) of the IOC sea-level monitoring facility hosted by the Flanders Marine Institute (VLIZ). The VLIZ website indicates the real-time status of stations and provides data at the frequency sampled by the tide gauge. This monitoring facility can provide an incentive for national participation as it allows suppliers to view instantly their data streams and identify faults, leading to better gauge maintenance.

Looking at a few numbers with regard to the GCN can indicate some of the challenges that continue to face the tide gauge networks. One of the reasons for establishing GLOSS was to improve the data flow into the PSMSL. Of the 290 GCN stations, VLIZ had received data from 195 at some point in the past. (It should be noted that VLIZ lists the status of over 700 stations in total. These numbers were extracted on 6 February 2014.) In the past 5 years, the PSMSL had received data for 205 of the 290 GCN stations. While these numbers seem roughly similar, 27 stations are contributing data to VLIZ but are not currently contributing values to the PSMSL. Some data suppliers may believe that supplying data to VLIZ is sufficient to feed the data into the other GLOSS data streams. However, the VLIZ data stream is not quality controlled, a task that is better handled by people with local knowledge. As quality control is a very labour-intensive activity, particularly for high-frequency data, there is currently little ability to extract the data from VLIZ into the other data streams. A second problem is that only 165 of the 205 stations from which the PSMSL had received data were also datum controlled (in the RLR set), making it impossible to construct long time series from the remaining 40 time series. These numbers indicate that the GLOSS network continues to need work, both in ensuring data makes it into all streams and in maintaining or implementing the minimum requirements of a GLOSS station for datum control.

## 3. Satellite altimetry

**Figure 6.**
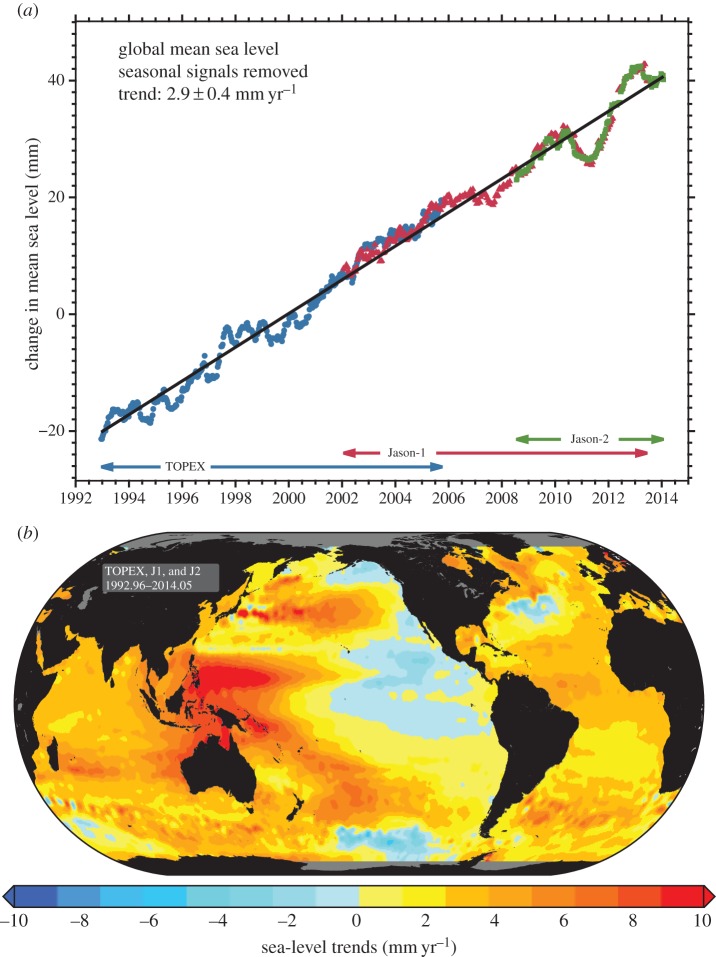
(*a*) Time series of the global average sea-level change from satellite altimetry (TOPEX/Poseidon, Jason-1 and Jason-2). Note that this time series has not been corrected for a contribution owing to GIA, estimated to be − 0.3 mm per year. (*b*) Map of the trend calculated from the altimetry time series since 1992. Images from the NOAA Laboratory for Satellite Altimetry.

Altimetric data, particularly from the TOPEX/Poseidon, Jason-1 and Jason-2 satellite missions, have provided a near-global coverage of the SSH change since the early 1990s. The global-average altimeter sea-level time series shows a rate of nearly 3 mm per year, after accounting for the associated GIA change (figure 6*a*). However, beyond the simple linear trend in the data, there is significant interannual variability. The global map of sea surface trends over the period also demonstrates large spatial variations away from this global average (figure 6*b*). The amplitudes and spatial patterns of these changes are driven mainly by dynamic processes. For example, the notable increase of SSH of 1 cm per year in the western Pacific is driven primarily by an intensification of Pacific trade winds [60]. This regional increase could represent a multi-decadal mode in the ocean [61]. Indeed, identifying and removing decadal and multi-decadal modes [62,63], as well as atmospheric wind and pressure effects [64,65], from the observations is becoming an increasingly common practice to help identify other underlying variability, such as a long-term rise.

Both globally and regionally, a change in the sea surface can result from a change in mass of the water column, or a change in density (local steric) caused by temperature and salinity changes [66]. Many mechanisms can lead to the mass and local steric changes, such as the movement of water (advection), surface fluxes of heat and freshwater, or mixing. However, simply using the local steric changes is an effective way to estimate the overall SSH change caused by these mechanisms. Indeed, the change in height of a water column given by steric changes reproduces much of the spatial pattern in sea-level change observed by altimetry [4]. To extract useful information about the mass change component of sea-level rise, which accounts for at minimum one-third (1901–1990) to over half (1993–2010) of the total of the global average [67], requires accurate assessment of the uncertainties in the local altimetric and steric trend estimates and an understanding of how systematic biases may influence the results.

An important first step in evaluating and using the data is to determine the significance of the estimated trend in the altimeter time series (either in the global average or at a particular location). While the magnitude of the trend from two time series can be identical, the uncertainties of the estimates can be very different depending upon the spectral content of the time series. If a large portion of the variability comes from interannual changes, then the time required to obtain a reliable estimate of a long-term trend will be significantly longer than if all variability apart from the trend is at periods shorter than a year. Hughes & Williams [8] recently applied spectral techniques to the altimetric time series to investigate the variability of the local observations, primarily as a tool to identify different dynamical processes. As part of that assessment, it was also possible to estimate the time span needed to measure a long-term trend with a 1 mm per year precision. For the global average, the required time span was found to be slightly more than 2 years, with 12 years being long enough to reduce the statistical error down to 0.1 mm per year. However, at individual locations in the ocean the 1 mm per year-level time span can range from 5 to 101 years. The longest time is associated with eddy variability in the Kuroshio extension off the east coast of Japan. This result highlights both the power of averaging to decrease the uncertainty in estimates of the trend as well as the fact that global maps of trends need to be viewed with these spatially varying uncertainties in mind.

While the variability in the time series places certain limits on our ability to make statements about the long-term trends, systematic factors also contribute to our uncertainty in the estimates of sea-level change. These systematic factors particularly highlight the need for simultaneous observations by other systems, as well as the need for a stable realization of the reference system for the measurements.

One important aspect of the altimetry is the need to correct for a variety of factors, including sea-state bias and wet tropospheric delay. These corrections vary between each satellite mission, must be independently evaluated and led to the requirement that the altimetry missions must overlap [68]. An important final check that errors in the corrections do not introduce errors into the altimetric long-term trend is to compare the altimeter time series with those from a selection of tide gauges distributed throughout the world [69]. In fact, the frequently reported uncertainty of 0.4 mm per year on the global trend is primarily associated with this check on altimeter drift [70]. This check is not perfect, in that not all of the tide gauges have GNSS measurements of vertical land (crustal) motion. This motion could introduce a systematic error into the analysis, though a recent study suggested that this globally averaged error should be less than 0.6 mm per year [71], which is also an independently estimated altimetric trend error [72]. However, the tide gauge comparison (which remains independent of altimeter processing streams) remains a crucial check that significantly larger erroneous trends are not present in the altimeter data. These figures are for global mean sea-level trend, which benefits from the fact that many regional sources of error, including orbit error, tend to cancel significantly in the global ocean average. Regional trend errors may be significantly larger at several millimetres per year [73].

The reference system is also vital to understanding sea-level change. One can think of a measurement of relative sea-level change, as measured at the coast by tide gauges, as the change in thickness of the ocean at a given location (figure 1). This logical extension of the tide gauge measurement allows us to visualize that relative sea level is a physical measurement. No matter in which reference frame we observe this thickness change, it must be the same. The same is not true for altimetry, which is referenced to the centre of mass of the whole Earth system (CM) on long time scales. On these longer time scales, the origin of the ITRF is designed to be the CM. The stability of this realization can be assessed by comparison with tide gauge or absolute gravity (AG) data (described more below), geophysical models (GIA or plate motion models), or geophysical inversions. A recent review has found that the ITRF is stable along each axis to better than 0.5 mm per year and has a scale error of less 0.3 mm per year [74]. While there are many aspects to the importance of a stable ITRF, a clear example for sea-level studies is the effect of geocentre motion on realizing the ITRF.

Geocentre motion is defined as the relative motion between the centre of mass of the whole Earth system (CM) and the centre of figure of the solid Earth (CF). One can understand that this motion will be important for the observing system when one considers that most methods of observing Earth deformation, such as GNSS, are all located on the solid Earth's surface and that the observation network then approximates CF, whereas satellites orbit around the CM. An easy way to visualize the motion is to think of a very large ice sheet on the North Pole. If that ice sheet were to melt, then mass would be transferred to the Southern Hemisphere, given the relative distributions of the continents and oceans. In this example, CM would move slightly southwards relative to CF. When combining satellite- and Earth-based observations, this motion must be accounted for. The challenge is in obtaining a stable estimate of this motion.

**Figure 7.**
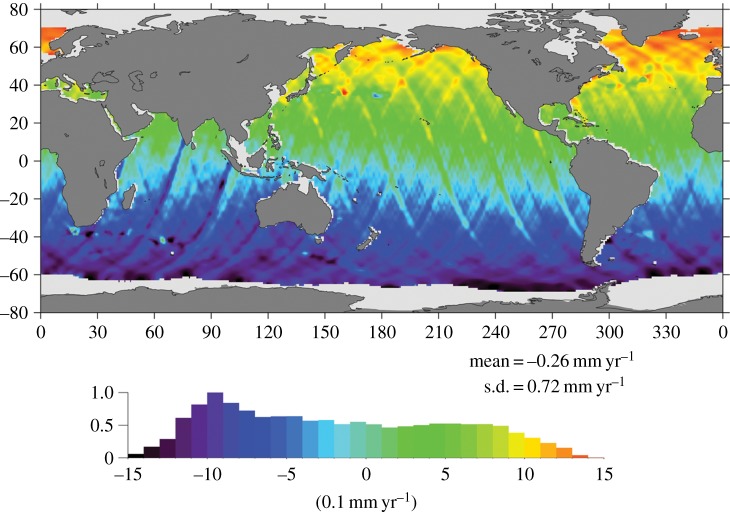
Difference between the trends calculated from the TOPEX/Poseidon altimetry mission using the default set of processing models and an updated set. (Figure 2 adapted from [75]. Copyright © 2007 American Geophysical Union.) While there are many features in the map, the most notable feature is the north/south gradient in the difference, which is a result of switching from CSR95, the default reference frame for the TOPEX/Poseidon products, to ITRF2005, the state-of-the-art reference frame at the time of the analysis. The normalized histogram on the bottom serves as the colour bar for the rate differences.

One of the best examples of the importance of geocentre motion for sea-level change is shown in figure 7. Beckley *et al*. [75] investigated the effect of reprocessing the TOPEX/Poseidon time series with a consistent and recent (at the time) set of analysis parameters. The resulting difference in the estimated sea-level trends between the new results and a previous analysis is shown in figure 7. While several features can be identified in this difference map, the most notable is south to north increase in the estimated trends, with the rates in the Southern Ocean decreasing by a millimetre per year and those in the northern Pacific and Atlantic Oceans increasing by a similar amount. This pattern is due to the switch of the reference frame from CSR95, which was the standard for the TOPEX/Poseidon products, to ITRF2005, the best ITRF realization at the time of the study. While this change had a small impact on the global average, the local changes were quite significant.

Currently, geocentre motion is only constrained in the ITRF by SLR data. In order to accurately determine satellite orbits, observatories measure the time it takes for a series of laser pulses to return from corner cube reflectors on the satellite as it passes overhead. Some of the most important targets, in terms of the reference frame, are the LAGEOS and other geodetic satellites, which are spherical satellites covered with reflectors. The combination and analysis of these tracking data made by the ILRS gives the best constraint on geocentre motion, earning its place of importance in the ITRF.

The UK has a long history of effort in SLR observations and the reference system more generally. Dr George Wilkins, at the Royal Greenwich Observatory at Herstmonceux, on the south coast of England, helped lead the Monitor Earth Rotation and Intercompare the Techniques (MERIT) project, which was initially proposed in 1978 [76]. Project MERIT eventually developed into the IERS, which is responsible for the ITRF. As part of the UK's contribution to this effort, SLR observations were undertaken at Herstmonceux, which is currently the NERC Space Geodesy Facility (NSGF). In 2013, NSGF celebrated 30 years of continuous SLR observations, during which time the ranging precision has improved by more than an order of magnitude to the current level of 1 mm.

Over this long history, NSGF has included other measurement techniques, such as GNSS since 1992, and AG since 2006 [77]. NSGF also has an active hydrogen maser clock, which greatly improves the timing for the SLR observations, as well as allowing it to contribute significantly to time transfer experiments and support of NASA's Lunar Reconnaissance Orbiter. Intercomparisons with other international SLR observing sites consistently show the NSGF to be among the most stable and least noisy sites [78], leading to a large weighting for NSGF measurements in the realization of the ITRF. Sites having several different observations are particularly important to the ITRF, as the ground ties between these different observational techniques allow for better, more stable solutions. In order to improve the stability of the reference frame, GGOS has created a new focus on establishing a set of core ground sites. At these core sites, SLR, VLBI, GNSS and DORIS measurements are all required. NSGF is currently accepted by GGOS as a new technology SLR site under the project, owing to its continued research and development of the technique and, as part of that development, is actively promoting a case to include VLBI capability. The addition of a VLBI antenna and perhaps a DORIS beacon would clearly allow NSGF, as a core GGOS site, to make an even larger UK contribution to future ITRF solutions.

This discussion regarding the geocentre motion, and its impact on sea-level estimates, helps to highlight one of the intrinsic uncertainties of trying to obtain ocean volume change from altimetry. Motion of the crust under the ocean, which will impact the estimate of the ocean's volume, remains unconstrained. Even assuming that ice sheets stopped exchanging water with the oceans in the past, GIA models predict that the ocean's crust should subside by 0.3 mm per year, the global average of a value that varies regionally, as the forebulges surrounding the locations of the former ice sheets collapse [34,79–81]. This (negative) contribution is frequently subtracted from the altimetry observation, increasing the estimated rate, to obtain a volume change, as in the value reported above. However, any other process driving crustal deformation in the ocean could also be responsible for net contribution over the ocean. Thus, there will always be some uncertainty in using the altimetry estimate as a measure of ocean volume change.

## 4. Gravity

One of the most significant improvements over the past decade is the measurement of the long-wavelength gravity field. Two complementary missions are responsible for this improvement: the GOCE [44] and the GRACE [45] satellite missions.

The GOCE mission was specifically designed to measure the geoid accurately enough to obtain dynamic topography when combined with altimetry, with the goal of obtaining 1 cm geoid accuracy from the longest length scales down to 100 km. The mission, launched in March 2009, returned to Earth in November 2013, lasting three times longer than expected. The longer lifetime allowed for additional measurement sessions at lower altitudes, finishing in an orbit of only 235 km above the Earth. The contours in figure 2 are of the 2002–2007 mean dynamic topography estimated using the TUM2013C geoid [18], which incorporates release 4 GOCE data, and the DTU10 mean sea surface [16,17]. These results are now of a quality that allows dynamic topography to be directly estimated from a combination of altimetry and the geoid down to length scales of about 100 km (half-wavelength). Typical formal errors of the release 4 solutions are 2–3 cm at this resolution, in line with predictions for a mission lasting until the end of 2012 [82], but realistic errors remain a subject of research and a substantial improvement is expected with release 5.

Temporal changes in the geoid are primarily caused by motion of water, whether in the ocean or stored on the continents in aquifers and groundwater, surface water, dams or snow and ice. While mass fluxes into the ocean can redistribute globally within a matter of days to weeks, reducing the magnitude of the geoid change, the localized changes of water storage on the continents causes geoid changes that are at least an order of magnitude larger than those over the ocean. In either case, most of the power in the time-varying geoid occurs at the longest wavelengths. Thus, while GOCE is accurate enough, especially when its data are combined with data from other satellite missions, to establish the static field needed to determine dynamic topography, another one to two orders of magnitude in accuracy at the longest wavelengths is needed in order to estimate the time-dependent field. This requirement led to the design of the GRACE satellite mission, a nominal 5 year mission launched in 2002 and still operating at the start of 2014. The release 5 of the data has obtained 1 mm accuracy in the geoid down to 400 km [83].

GRACE provides the most direct estimate of the mass change of the ocean. This solution can be obtained in two ways: either looking at the mass change over the oceans directly [84–87] or obtaining the complementary measurement of the mass change over the continents [88,89]. These results suggest a mass flux into the ocean over the GRACE time period, where the trend in ocean mass is equivalent to a sea-level rise of 1.1±0.6 mm per year [90]. While GRACE data provide the best observation, the estimate relies on careful data analysis, as well as auxiliary observations and models that can affect the results. The averaging over the oceans must necessarily exclude coastal regions owing to the large continental geoid changes that can contaminate the ocean data [91].

Two auxiliary observations and models are also quite important: geocentre motion and ongoing Earth deformation. Given the asymmetric distribution of the oceans, if water is transferred, on average, to the oceans from the continents, then there should be net motion of water mass to the Southern Hemisphere. However, GRACE orbits about the CM, and thus is unable to directly observe this (the degree-one spherical harmonic) net mass motion. If one were to completely ignore this geocentre motion, in combination with smoothing commonly applied to the data to reduce short-wavelength errors, then the observation of mass loss from Greenland could be underestimated by as much as 60% [92]. One possible solution is to add the geocentre contribution through an indirect calculation using the GRACE estimates [93]. Ongoing Earth deformation owing to GIA causes mantle mass to flow, on average, from under the oceans to under the continents. Because of the greater density of the solid Earth compared with water, the relatively small motion can result in a large contribution to the apparent ocean mass change if it is wrongly assumed to be owing to water thickness change. Currently, the only way to estimate this contribution to GRACE measurements is from GIA forward models. This correction to the global trend in mass-related sea level can be quite significant, over 1 mm per year [94–96], and the uncertainty can also be large, up to 0.4 mm per year [34].

GRACE also allows us to identify the causes of interannual variability present in the sea-level record. Altimetry (figure 6*a*) shows a significant decrease in mean sea level from 2010 to the middle of 2011, with sea level rapidly recovering in 2012. GRACE demonstrated that, during this period, there was a large increase in water storage in northern South America and particularly Australia [97]. Further study indicated that the longevity of the anomaly could be attributed to water storage in closed drainage basins in Australia [98]. Particularly with the new release 5 data, it is also possible to examine regional signals in ocean bottom pressure (OBP) observed from GRACE [99]. However, because of the relatively small amplitude of these signals compared with the noise in the monthly estimates, earlier results primarily focused on large basin averages, such as interannual mass exchange between the Atlantic, Pacific and Indian Oceans [100], or the largest bottom pressure signals. GRACE has been used to examine the seasonal variability in different regions associated with the Antarctic circumpolar current, finding reasonable agreement with ocean model results [101,102]. The North Pacific has a large bottom pressure (mass) signal, with suggestions of a correlation with El Niño/Southern Oscillation (ENSO) variations in the subpolar gyre [103], and the region has also experienced a significant trend in ocean mass, of nearly 1 cm per year, between 2003 and mid-2007 [104].

While GRACE provides a good observation of the global average ocean mass change, given the possible systematic uncertainties described earlier, an additional check on the result, similar to the verification provided by tide gauges on the altimetric-derived rate, would be beneficial. Measurements of OBP in the central, equatorial Pacific may provide such a check [105]. A Monte Carlo simulation based on the spectra of ocean-model time series from this region suggests that 95% of all local sea-level trends will be less than 0.28 mm per year over 10 years, owing to the relatively small interannual dynamic variability in this region. In addition, no matter from which continental region mass enters the ocean, this area would observe nearly the same mass change, albeit biased uniformly high. Thus, the region should be ideal for observing long-term changes in ocean mass. Currently, however, OBP recorders suffer from nonlinear drift that prevents their observations from being used to derive trends. However, Hughes *et al.* [105] did demonstrate that the concept was able to retrieve an estimate of the ocean mass annual amplitude that was in good agreement with other observations. Thus, if OBP recorder technology could be improved to remove the drift [106], then this system could provide an ideal auxiliary measurement to the satellite-derived estimates.

## 5. Crustal motion

As tide gauge observations record the sea surface relative to the nearby land, local vertical land motion can be a significant contribution to the measured sea-level change. As illustrated in §1, crustal motion plays an important role in relative sea-level change in some areas, including the UK. The influence of the presence of the British–Irish ice sheet during the last glacial cycle is still a large contributor to the spatial variations in sea-level rise observed around the UK coasts. (It should be noted that the Fennoscandian and Laurentian ice sheets also play an important, albeit more spatially uniform, role in the ongoing GIA response around the UK [107].) The crustal motion associated with the return flow of mantle material from the peripheral regions to under the former loading centre contributes to spatial gradients in vertical crustal motion along the coast. Besides these large length-scale geodynamic changes, there may also be much more localized crustal motion. For example, a tide gauge may be located on a pier that is subsiding. On larger scales, small regions may be subsiding owing to a variety of causes, for example groundwater extraction and mining. Given that coastal infrastructure is built on this crust, understanding past observations and planning for future sea-level rise must consider these motions. There are a variety of observational techniques that can be used, and we describe two in the following.

GNSS networks, particularly where receivers are installed as part of the tide gauge or monitor the motion of the tide gauge bench mark, can assess the vertical land motion contribution. Removing this estimated vertical land motion effectively transforms the tide gauge measurement into a geocentric sea-level, or sea surface, measurement. Because the crustal motion is generally much more localized, and of larger amplitude, than the changes to the sea surface due to geoid changes (compare figure 3*f* with figure 3*d* for an example from a GIA model), removing a GNSS-derived rate from the tide gauge observations can produce a more regionally uniform estimate of sea-level change [108,109]. Using a criterion of a minimum of 60 year record length and 70% completeness for the tide gauge records, 61 tide gauge stations were identified near GNSS stations in a recent GNSS solution designed for use in a tide gauge analysis. Removing these GNSS-derived rates reduced the dispersion in the individual tide gauge records from 2.7 to 1.0 mm per year [71]. When correcting a tide gauge rate derived from many decades of data with a GNSS-derived rate from a much shorter time period, care must be taken that the more recent trend in crustal motion is expected to be representative of centennial-scale deformation [71]. In addition, as many GNSS installations are not co-located with the tide gauge, one must assume that the crustal motion is the same between the tide gauge and the GNSS station. We return to this assumption below.

**Figure 8.**
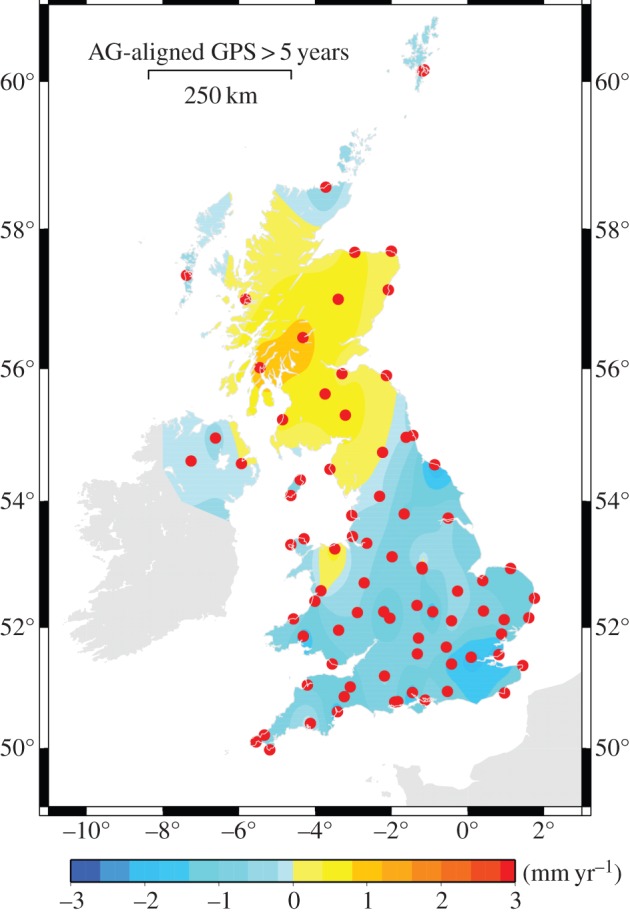
Estimate of the vertical land motion across the UK derived from a GNSS solution provided by the NERC British Isles continuous GNSS Facility (BIGF). Dots indicate the positions of the GNSS stations that contributed to the solution. The map of vertical land motion is an interpolation from the estimates at these sites.

In the UK, GNSS data are archived at the NERC British Isles continuous GNSS Facility (BIGF). The longest time series in the archive are around 17 years long, but these are from a fairly sparse set of locations. More recently, the number of available stations has increased to over 150 [110]. Analysis of the data provides a fairly compelling picture of GIA across the UK, as shown in figure 8. Regions under the centre of the former ice sheet are uplifting, whereas the surrounding regions are subsiding. As the spatial coverage and time span of the observations has improved, these inferred maps [110,111] of crustal motion have become more similar to the predicted GIA signal [107,112]. However, there is a continued requirement to collect the GNSS data to improve the solution and better understand the behaviour at some sites. For example, data from Sheerness are not included in figure 8 owing to the nonlinear behaviour of the record. Several other records were also excluded owing to their disagreement with nearby sites. In addition, continued collection of the data can assist in implementing improvements to the GNSS-processing models and techniques that also contribute to a better estimate of spatial pattern of vertical land motion.

The most recent GLOSS implementation plan requires GNSS receivers as close as possible to tide gauge benchmarks of the GCN sites [59]. To assist in generating GNSS solutions appropriate for use in sea-level analysis, the IGS established the GNSS Tide Gauge Benchmark Monitoring (TIGA) pilot project in 2001 [113], which was made a working group in 2010. TIGA works closely with GLOSS, and currently Système d’Observation du Niveau des Eaux Littorales (SONEL) serves as the GLOSS GNSS data assembly centre and the primary TIGA data centre. On their websites, direct links exist between corresponding observations at the PSMSL and SONEL, and SONEL has created an interactive map that allows one to quickly switch between the tide gauge and geocentric rates at locations where a GNSS solution is available. SONEL had data from 167 GNSS sites near GCN tide gauges, and its recent global solution had 282 GNSS solutions near (within 15 km) tide gauges [71]. Looking beyond the GCN, King *et al*. [114] found that only one-third of tide gauges used in global sea-level reconstruction are near GNSS stations.

A disadvantage of GNSS estimates of crustal motion is that they are a very local measurement. While site selection for the placement of permanent stations attempts to eliminate the possibility of sampling anomalous regional motion, there are some instances where an observation is only applicable to a few kilometres or less. Thus, another measurement that gives a comprehensive regional estimate of crustal motion would be beneficial in determining the connection between the GNSS-estimated rate and the tide gauge observation. Persistent scatterer interferometry (PSI) [115] can provide such a method. Several studies have demonstrated the utility of using PSI to look at differential signals within port cities, such as Los Angeles, CA [116], Venice, Italy [117], and Alexandria, Egypt [118]. The technique was also used in a comprehensive assessment of the Thames estuary region to determine highly resolved estimates of subsidence and uplift for use in flood risk management [119]. Another study used PSI measurements around four UK tide gauges (Liverpool, North Shields, Sheerness and Newlyn), where the technique was particularly successful in more dense urban environments such as Liverpool and North Shields [120]. At these locations, the results demonstrated that, although there was visible localized ground motion within the image, the locations of the tide gauge and the GNSS stations were unaffected. Currently, the limited availability of data prevents widespread use of this technique, but this situation will be dramatically improved with data from Sentinel-1 [121].

An important component of the UK GNSS studies was the contribution of AG measurements [122]. As the crust subsides, gravity will increase as one moves closer to the Earth's centre. Thus, AG measurements can provide an independent measurement of the vertical land motion in the region, with no reliance on reference frame stability, though other assumptions concerning length scales and processes are required. From 1995/1996 until 2010, AG measurements were made in the UK at Newlyn, Aberdeen and Lerwick. These observations helped constrain the early GNSS solutions, where vertical rates could vary significantly depending upon processing [111]. This constraint has become less important given recent improvements in GNSS processing and increased stability of the reference frame. However, AG measurements still provide an important independent check of the vertical rates estimated from GNSS observations.

In the UK, the Environment Agency (EA) has been responsible for supporting the tide gauge network. Unfortunately, starting in 2010, the EA could no longer fund the maintenance of the GNSS at the tide gauges or the collection of the AG measurements. In the case of the GNSS, NERC supported BIGF to upgrade the equipment in 2013 and to continue collection and processing of the GNSS data at the tide gauges. However, their maintenance and long-term status is not guaranteed, which places the UK in danger of no longer being able to satisfy the GLOSS station criteria, as well as decreasing the scientific value of the simultaneous time series. In addition, losing the AG measurements has meant losing an important independent check on the vertical motions across the UK. This provides an example of the importance of reliable long-term funding for sustained observations.

## 6. The future

Improvements in the observations contributing to the reference system allow many of these disparate measurements to be integrated for the first time, finally answering some of the questions that led to the initial collection of the mean sea-level data. While Professor Witting was interested in the mean dynamic topography present in the Baltic, the character of mean dynamic topography along the US East Coast led to a disagreement between oceanographers and geodesists. Levelling combined with mean sea-level observations from tide gauges suggested that mean dynamic topography increases from south to north along the coast [123], whereas oceanography would suggest that it should decrease [124], though the mechanism of that decrease is subtle and still a subject of debate. Levelling results still demonstrate this increase from south to north. However, correcting the mean sea-level observations referenced to ellipsoidal heights using GNSS with the geoid heights from the newest models that use GRACE and GOCE data shows a decrease from south to north. This new result agrees well with mean dynamic topography results generated from a number of ocean models [125]. The agreement demonstrates that modern geodetic measurements, combined with ocean models for mean dynamic topography corrections, are of sufficient accuracy to allow for a unification of different national datums, enabling world height unification. This is a major advance that would not have been possible even 10 years ago.

Beyond improvements that result from improved observations contributing to the reference system, other observation systems have evolved that will allow us to better understand sea-level change in the future. One of the most important is the ability to use altimetry results closer to the coasts. Classical altimetry methods were geared to the open ocean, and a number of issues (contamination of the radar or radiometer footprints by land returns, time taken for the tracking system to lock onto the ocean after passing over land) meant that there was a wide band surrounding the coast, typically tens of kilometres, in which sea-level measurements were corrupted or absent. This limited the ability to tie these observations of the open ocean with the tide gauge data at the coasts. However, there have been a number of improvements, particularly in tracking and algorithms to eliminate land contamination, as well as the new synthetic aperture radar (SAR) mode technology on Cryosat-2 and most future altimeters, which mean that it is now possible to make good measurements much closer to the coast (closer than 1 km in regions where the satellite track is perpendicular to the coast, up to 4 km in less favourable geometries and wave conditions [126]). The SAR concept is taken further in the proposed swath altimeter missions, such as Surface Water, Ocean Topography (SWOT), which aims to extend altimeter measurements to a finely resolved off-nadir swath to either side of the satellite track [127].

Improvements in time-dependent geoid determination, and hence in measuring global mass movements, can also be expected. A number of simulations have demonstrated that significant gains can be made by flying two pairs of GRACE-like satellites in complementary orbits, and the potential addition of a future generation of gradiometer instruments could improve the spatial resolution of such a system still further [128]. Improvements in satellite–satellite tracking are expected, and improving clock technology is also important. There is still a long way to go before we exhaust the possible improvements in time-dependent satellite gravity measurements, and only time will tell how far that improvement can go.

A substantial improvement on the static geoid measurement from GRACE plus GOCE would be very difficult from space-based measurements, requiring an improvement in the accuracy of gravity gradiometers by several orders of magnitude. GOCE currently gives 2 cm accuracy on length scales (wavelengths) of 250 km. This level of spatial resolution will miss significant geoid variations caused by topographic variations over tens of kilometres, which may be particularly important for resolution of boundary currents and in the coastal zone. However, it would be possible to make *in situ* point measurements of the geoid, analogous to a GNSS measurement of crustal position, if clock technology could be improved. To measure a geopotential difference to an accuracy equivalent to 1 cm by comparing two clocks, they must be accurate at the 10^−18^ level [129]. Comparing these measurements represents a real challenge, but global time transfer at this accuracy is an aim of the proposed ESA Explorer satellite mission STE-QUEST [130]. A recent laboratory experiment was able to measure a height change of 33 cm using optical clocks [131], a height difference comparable to the present-day point-wise accuracy of the geoid based on satellite measurements, with the error being dominated by the geoid variation on length scales shorter than those resolved by the satellite data (omission error).

A recent development in GNSS analysis is the use of multi-path, typically an error source, to allow GNSS to effectively serve as a tide gauge [132–134]. Multi-path is the combined measurement of the direct signal and reflections, scatterings or diffractions from the site-specific environment (primarily) close to the antenna. Recent studies [132–134] have shown that these multi-path effects can be used to accurately measure environmental signals such as soil moisture, snow depth and SSH. By design, these sea-level measurements will be with respect to the terrestrial reference frame, and the GNSS station will also provide a simultaneous estimate of vertical land motion. If a GNSS instrument suitably near a conventional tide gauge can also measure SSH, then GNSS reflection studies can be used to level between the two instruments without the need for conventional techniques. In some cases, such as inaccessible coastlines along cliffs, GNSS ‘tide gauges’ could also be installed where a conventional gauge could not. With over 10 000 GNSS stations already continuously operating around the world, if only a small fraction of these are in an appropriate location, then the number of ‘tide gauge’ stations could effectively increase without the need for additional networks or infrastructure.

As we continue to use sea-level data to understand the mechanisms contributing to regional sea-level change, GNSS can be used reliably to remove the vertical land motion from tide gauge data for comparison with altimetry data. However, a global combination of GNSS and PSI satellite measurements could add a significant amount of socially relevant information to the altimetry observations. Indeed, the traditional separation of domains of responsibility into ‘ocean’ and ‘land’ means there are logistical difficulties to effective communication across the coastal boundary. The increasing importance of understanding the interaction between flooding owing to coastal events and that owing to intense rainfall, and the need to incorporate results of global ocean and climate models into local impact assessments and predictions, requires that this effort be made.

This review has attempted to show how a number of sustained observations, including those beyond the widely recognized tide gauge and altimetry measurements, are needed to monitor and understand sea-level change. By comparison and integration of these observations, we can obtain better estimates of the causes of these changes and uncertainties in the measurements. Owing to the global nature of both the problem and the data sources, this effort will continue to require international cooperation, coordination and financial support.
